# Highly Sensitive Whole-Cell Biosensor for Cadmium Detection Based on a Negative Feedback Circuit

**DOI:** 10.3389/fbioe.2021.799781

**Published:** 2021-12-03

**Authors:** Guangbao Zhang, Shuting Hu, Xiaoqiang Jia

**Affiliations:** ^1^ Department of Biochemical Engineering, School of Chemical Engineering and Technology, Tianjin University, Tianjin, China; ^2^ Frontier Science Center for Synthetic Biology and Key Laboratory of Systems Bioengineering (MOE), School of Chemical Engineering and Technology, Tianjin University, Tianjin, China; ^3^ Collaborative Innovation Center of Chemical Science and Engineering (Tianjin), Tianjin, China

**Keywords:** cadmium detection, negative feedback amplifier, sensitivity, specificity, whole-cell biosensor

## Abstract

Although many whole-cell biosensors (WCBs) for the detection of Cd^2+^ have been developed over the years, most lack sensitivity and specificity. In this paper, we developed a Cd^2+^ WCB with a negative feedback amplifier in *P. putida* KT2440. Based on the slope of the linear detection curve as a measure of sensitivity, WCB with negative feedback amplifier greatly increased the output signal of the reporter mCherry, resulting in 33% greater sensitivity than in an equivalent WCB without the negative feedback circuit. Moreover, WCB with negative feedback amplifier exhibited increased Cd^2+^ tolerance and a lower detection limit of 0.1 nM, a remarkable 400-fold improvement compared to the WCB without the negative feedback circuit, which is significantly below the World Health Organization standard of 27 nM (0.003 mg/L) for cadmium in drinking water. Due to the superior amplification of the output signal, WCB with negative feedback amplifier can provide a detectable signal in a much shorter time, and a fast response is highly preferable for real field applications. In addition, the WCB with negative feedback amplifier showed an unusually high specificity for Cd^2+^ compared to other metal ions, giving signals with other metals that were between 17.6 and 41.4 times weaker than with Cd^2+^. In summary, the negative feedback amplifier WCB designed in this work meets the requirements of Cd^2+^ detection with very high sensitivity and specificity, which also demonstrates that genetic negative feedback amplifiers are excellent tools for improving the performance of WCBs.

## 1 Introduction

Cadmium (Cd) is a heavy metal without a physiological function and considerable toxicity (Sinicropi et al., 2010; Friberg et al., 2019). Over the past century, many different forms of cadmium exposure have been identified, among which groundwater cadmium pollution is a severe global health problem (Rahimzadeh et al., 2017). The continuing cadmium contamination is related to its application in industry as a corrosive agent as well as its use in polyvinyl chloride (PVC) products, pigments, and Ni-Cd batteries (Genchi et al., 2020). Long-term exposure to cadmium can cause a variety of diseases, including cancer (Saha et al., 2016; Zhang and Reynolds, 2019). Cadmium pollution in the environment is a serious international problem, affecting the health of hundreds of millions of people worldwide (Elonheimo et al., 2021). Due to its toxicity and harm to human health, cadmium detection requires low-cost, sensitive environmental monitoring equipment.

The World Health Organization (WHO) has set the safe allowable level of cadmium in drinking water at 27 nM (0.003 mg/L). The EPA recommends 1 × 10^−3^ mg/kg/day and 5 × 10^−4^ mg/kg/day as reference doses for human exposure to Cd^2+^ in food and water. However, the cadmium concentrations released into water from industrial activities are much higher than the prescribed limits, causing environmental contamination and increasing hazards to human health (WHO, 2011; Ashizawa et al., 2012). At present, atomic absorption spectrophotometry, dithizone spectrophotometry, atomic fluorescence spectrometry, and anodic dissolution voltammetry are the globally approved techniques for measuring cadmium concentrations (Ure et al., 1978; Iu et al., 1979; Albert et al., 1992; Hou et al., 2001; Feng and Liu, 2010; Rosolina et al., 2015; Santos et al., 2015; Kumar et al., 2017; Zhou et al., 2018). However, the pre-processing and analysis of materials using these procedures frequently requires complicated and expensive instruments and experienced specialists, making them challenging to utilize in the field. Accordingly, biosensors based on enzymes, antibodies, and microbial cells have sparked interest for detecting cadmium in drinking water (Van Der Meer and Belkin, 2010; Bereza-Malcolm et al., 2015).

In recent years, whole-cell biosensors (WCBs) have received increasing attention as a precise and sensitive way of detecting hazardous heavy metal ions. Heavy metal resistance operon regulatory elements (such as transcriptional regulators and their cognate promoters) are frequently connected to reporter genes that generate a measurable output (such as fluorescence, luminescence, or enzyme analysis), so that the reporter’s signal intensity is proportional to the concentration of the heavy metal to be detected. A relatively well-studied cadmium-resistance operon was found in P. putida 06909, which contains CadR (a transcriptional regulator) and CadA (a P-type ATPase metal efflux pump) (Lee et al., 2001). In the absence of Cd^2+^, CadR binds to the CadR binding site within the P_*cad*_ promoter, which prevents RNA polymerase from recognizing the promoter P_*cad*_ bidirectional and blocks the transcription of *cadR* and *cadA*. In the presence of Cd^2+^, CadR binds to Cd^2+^ and alters the local structure of the promoter, stimulating *cad* gene transcription and eliminating cadmium from the cell (Ma et al., 2009; Summers, 2009; Liu et al., 2019). CadR promoters and regulators have been utilized to construct cadmium WCBs in a variety of microbial hosts (Sørensen et al., 2006; Van Der Meer and Belkin, 2010; Su et al., 2011). However, when used for cadmium detection below the WHO limit, poor sensitivity and specificity are serious concerns (Sayut et al., 2006; Close et al., 2012; Kim et al., 2016; Bereza-Malcolm et al., 2017).

In this study, we used *P. putida* KT2440, which naturally contains the cad operon, as a host to construct cadmium WCBs with improved performance through genetic circuit engineering techniques. The sensitivity of the biosensor is closely related to the relative concentration of the receptor (CadR) and ligand (Cd^2+^). Accordingly, the detection limit can be increased by adjusting their respective concentrations in the cell. A high-gain transcription amplifier based on the tetracycline repressor (TetR) negative feedback was designed as the amplification module to adjust the intracellular concentration of CadR, thereby increasing the expression of the reporter protein MCherry in the output module to increase its dynamic. Negative feedback loops are prevalent in nature, and negative feedback mediation mechanisms are widely recognized (Savageau, 1974). Such loops have been utilized to increase the sensitivity of WCBs to a range of analytes, including antibiotics, heavy metals, and other contaminants (Bertram and Hillen, 2008; Wang et al., 2015a; Zhang et al., 2020). This work introduces a negative feedback loop that incorporates TetR self-regulating elements into cadmium WCBs to improve the sensitivity and specificity of WCBs for the first time. This paper presents a comparison of designs with and without negative feedback amplifiers, which offers valuable insights for efficient combined circuit forms of functional and modulation modules in microbial sensors based on negative feedback amplifiers and the future development of WCBs.

## 2 Materials and Methods

### 2.1 Bacterial Strains, Reagents, and Culture Conditions

The intended WCBs were constructed and characterized in *P. putida* KT2440. LB broth (10 g/L peptone, 5 g/L NaCl, 5 g/L yeast extract) was used to culture the cells, and 50 mg/L kanamycin (Kan) was added where required. The same medium was used to make solid plates, with 1.5 percent (wt/vol) agar added. Unless stated otherwise, all experiments were carried out at 30°C.

The PCR reagents, restriction endonucleases, and the Basic Seamless Cloning and Assembly Kit were purchased from TransGen Biotech (China). CdCl_2_, Pb(NO_3_)_2_, ZnCl_2_, CuCl_2_, and NaAsO_2_ were purchased from Shandong Western Chemical Industry Co. Ltd., China. Anhydrotetracycline hydrochloride (aTc, purity ≥ 98%) was purchased from Aladdin (China). PCR primer synthesis and sequencing were performed by Genewiz (China).

### 2.2 Design and Construction of WCBs

The *cadR* coding sequence and the P_*cad*_ promoter that controls its expression were amplified from the genome of *P. putida* KT2440 (GenBank: AE015451.2). The plasmid pAM1 served as a template for the amplification of the *mcherry* gene, codon optimized for *P. putida*. The genome of *Escherichia coli* strain DH5α (NCBI Reference Sequence: NZ CP026085.1) served as a template for the amplification of *tetR*, which was then codon-optimized for *P. putida*. The TetR repressible promoter P_*lteto1*_ was synthesized following the BioBrick standard (http://biobricks.org). A terminator (BBa_B0015) was synthesized based on the iGEM Registry (http://parts.igem.org/Catalog) and was used to terminate gene transcription in all cases. All genes were synthesized and optimized by Genewiz (China) (Supplementary Table S1). Cadmium WCB gene circuits were constructed in the broad-host-range shuttle plasmid pBBR1MCS-2 (Supplementary Figures S1, S2), and PCR/gel electrophoresis and Sanger sequencing were used to confirm each of the genetic circuits.

### 2.3 Growth Curves of the WCB Strains

The growth curves of the constructed WCBs were examined at different Cd^2+^ concentrations to understand the harmful effects of Cd^2+^ on the engineered strains. A CdCl_2_ solution was added to the shake flasks to achieve a final concentration of 0, 0.1, 1, 10, 100, 200, 300, 400, 500, or 600 μM, and aTC solution was added to the shake flask to achieve a final concentration of 400 ng/ml. They were incubated at 30°C and 220 rpm. Before the addition of Cd^2+^, samples were taken every hour. When the OD_600_ value reached 0.6–0.8, the addition of Cd^2+^ was started, after which the samples were taken every 2 hours, and the OD_600_ was measured using a UV spectrophotometer.

### 2.4 Measurement of the Response Time of WCBs

In terms of practical use, the response time of WCBs is critical. When the bacterial solution’s OD_600_ reached 0.6–0.8, CdCl_2_ solution was added to the test tubes to achieve final concentrations of 0, 0.01, 0.02, 0.04, 0.05, 0.1, 1, or 10 μM, and aTC solution was added to a final concentration of 400 ng/ml. The test tubes were incubated at 30°C and 220 rpm. Every 2 h, samples comprising 200 μL of the bacterial culture were transferred to a 96-well plate, and the RFU and OD_600_ of the samples were determined using a microplate reader.

### 2.5 Dose-Dependent Response to Cadmium

The whole-cell sensor’s fluorescence response was studied at various Cd^2+^ concentrations. Final concentrations of 0, 0.00001, 0.0001, 0.001, 0.01, 0.02, 0.04, 0.05, 0.1, 1, or 10 μM Cd^2+^ and 400 ng/ml aTC solution were added when the OD_600_ of the bacterial solution reached 0.6–0.8, followed by incubation at 30°C and 220 rpm. After 8 h, 200 µL of the bacterial solution was transferred to a 96-well plate, and the RFU and OD_600_ values were determined using a microplate reader.

### 2.6 Specificity of the WCBs

The specificity for cadmium, in addition to the sensitivity and output signal intensity of WCBs in reaction to cadmium, is an essential element in the evaluation of WCBs. Cd^2+^, Pd^2+^, Zn^2+^, Cu^2+^, and As^3+^ solutions with final concentrations of 0.01, 0.1, 1, or 10 μM and a final concentration of 400 ng/ml aTC were added to a bacterial solution with an OD_600_ of 0.6–0.8 and incubated in shake flasks at 30°C and 220 rpm. After 8 h, 200 μL of the bacterial solution were pipetted to a 96-well plate, and the RFU and OD_600_ values were measured using a microplate reader.

### 2.7 Fluorescence Measurement

Fluorescence intensity was measured using a microplate reader (infinite 200Pro; TECAN, Switzerland), with 200 μL of the sample per well of a 96-well microtiter plate. The optical density was measured at 600 nm and the fluorescence intensity was measured using excitation/emission wavelengths of 580/610 nm, respectively. All tests were carried out in triplicates, with the unmodified *P. putida* KT2440 strain as a negative control (Jia et al., 2019).

The formula FIR = AFU/BFU was used to calculate the fluorescence induction rates (FIRs). The relative fluorescence value (RFU) was divided by the sample absorbance to obtain the fluorescence value (AFU). The fluorescence value of the control (BFU) was defined as the relative fluorescence value (RFU) of the unmodified *P. putida* KT2440 strain (negative control) divided by its absorbance. The control assay fluorescence value (BFU) was set to 1.0, and the other samples were normalized to the control assay fluorescence value (BFU).

### 2.8 Important Indicators of WCBs

The response time of the biosensor for Cd^2+^ was determined as the time at which the fluorescence response changed substantially (*p* < 0.05) at low Cd^2+^ concentrations (≤0.05 µM). The detection limit of the biosensor was established as the Cd^2+^ concentration that induced a significant change in fluorescence response (*p* < 0.05) (Liao et al., 2006; Hou et al., 2015; Bereza-Malcolm et al., 2017). The sensitivity of the biosensor was determined by the slope of the linear detection curve of the fluorescence response to Cd^2+^ concentration (Pola-López et al., 2018; Guo et al., 2019). The specificity of the biosensor was determined by the lowest ratio of the biosensor’s fluorescence response to Cd^2+^ at 0.1 µM to the fluorescence response to other heavy metal ions at 10 µM.

### 2.9 Analyses of Spiked Sample

To remove impurities and bacteria, the initial river water samples (collected from Longfeng River, Wuqing District, Tianjin) were filtered using a 0.22-μm pore-size membrane. To assess whether additional ions or complexes in the river water have an effect on the biosensor, LB medium was prepared directly from river water samples with 50 mg/L kanamycin for bacterial culture. Various concentrations of cadmium (0.01, 0.027, 0.045, 0.050 μM, all samples in triplicate) were added to 10 ml aliquots of bacterial culture in the test tubes and incubated until the OD_600_ of the bacterial culture reached 0.6–0.8. At the same time, the medium for bacterial growth was produced using ultrapure water, and a standard curve was created by adding a series of cadmium concentrations (0.01, 0.02, 0.04, 0.05 μM) (Supplementary Figure S3). To replicate a field test setting, the trials were carried out at room temperature. The standard curve equation was then used to calculate the concentration of cadmium ions in river water samples. Similarly, based on the 6 h time-dependent fluorescence response, we tested the sensor’s detection performance at medium and high concentrations of cadmium. Various concentrations of cadmium (5, 10, 50, 100, and 200 μM, all samples in triplicate) were added to 10 ml aliquots of bacterial culture in the test tubes and incubated until the OD_600_ of the bacterial culture reached 0.6–0.8.

## 3 Results

### 3.1 Design and Construction of Biosensor

**FIGURE 1:**
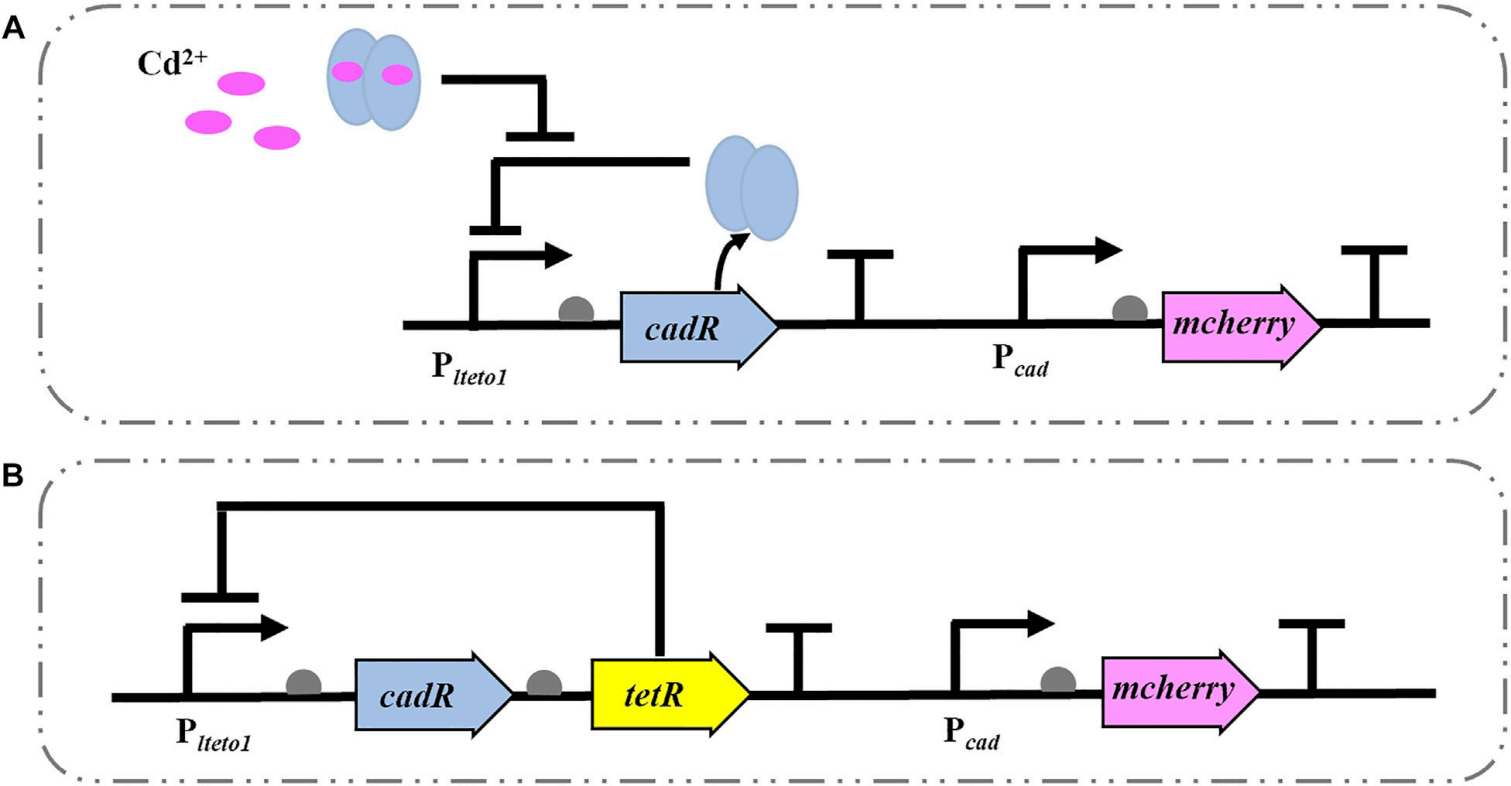
Schematic diagram of a Cd^2+^ WCB without **(A)** and with negative feedback **(B)**. **(A)** A typical Cd^2+^ WCB CM consists of the promoter P_*lteto1*_, the regulator cadR, cadR-regulated promoter P_*cad*_, and the reporter gene mcherry. **(B)** The negative feedback WCB TCM additionally incorporates a negative feedback amplifier that uses the tetracycline repressor protein TetR to regulate the specific promoter P_*lteto1*_.

The Cd^2+^ WCBs without (CM) and with (TCM) negative feedback were constructed in P. putida KT2440, as illustrated in Figure 1. The biosensor CM is based on the cadRA operon of *Pseudomonas* putida with its intrinsic Cd^2+^ regulation mechanism. The P_*lteto1*_ promoter (constitutively ON and repressed by TetR.), detection element and terminator BBa_B0015 (P_*lteto1*_-*cadR*-T), and cadmium-inducible promoter (P_*cad*_) gene were coupled to the reporter element red fluorescent protein gene mcherry to construct a gene circuit (P_*lteto1*_-*cadR*-T-P_*cad*_-*mcherry*-T) (Figure 1A). The intensity of the output signal of the red fluorescent protein mCherry is proportional to the concentration of the inducer Cd^2+^. As shown in Figure 1B, based on the biosensor CM, the TetR (inhibited by the addition of tetracycline or its analog, aTc), which regulates the intensity of the specific promoter P_*lteto1*_, was added to construct the biosensor TCM for transcriptional control by regulating the concentration of the intracellular receptor (cadR) (Wang et al., 2015b). When aTc is not present, TetR binds to the specific promoter P_*lteto1*_ and blocks the expression of the downstream genes. When aTc is added, it reduces the intensity of TetR inhibition at the specific promoter P_*lteto1*_. The resulting TetR continues to reduce the intensity of promoter P_*lteto1*_, keeping the intracellular CadR protein at a low level, forming a negative feedback loop that acts as a negative feedback amplifier for Cd^2+^ WCBs, enhancing the expression of the biosensor’s mCherry output signal. (Wang et al., 2015b). When aTc is added, it reduces the intensity of TetR inhibition at the specific promoter P_*lteto1*_. The resulting TetR continues to reduce the intensity of promoter P_*lteto1*_, keeping the intracellular CadR protein at a low level, forming a negative feedback loop that acts as a negative feedback amplifier for Cd^2+^ WCBs, enhancing the expression of the biosensor’s mCherry output signal.

### 3.2 Growth Curves of Whole-Cell Sensor Strains

**FIGURE 2:**
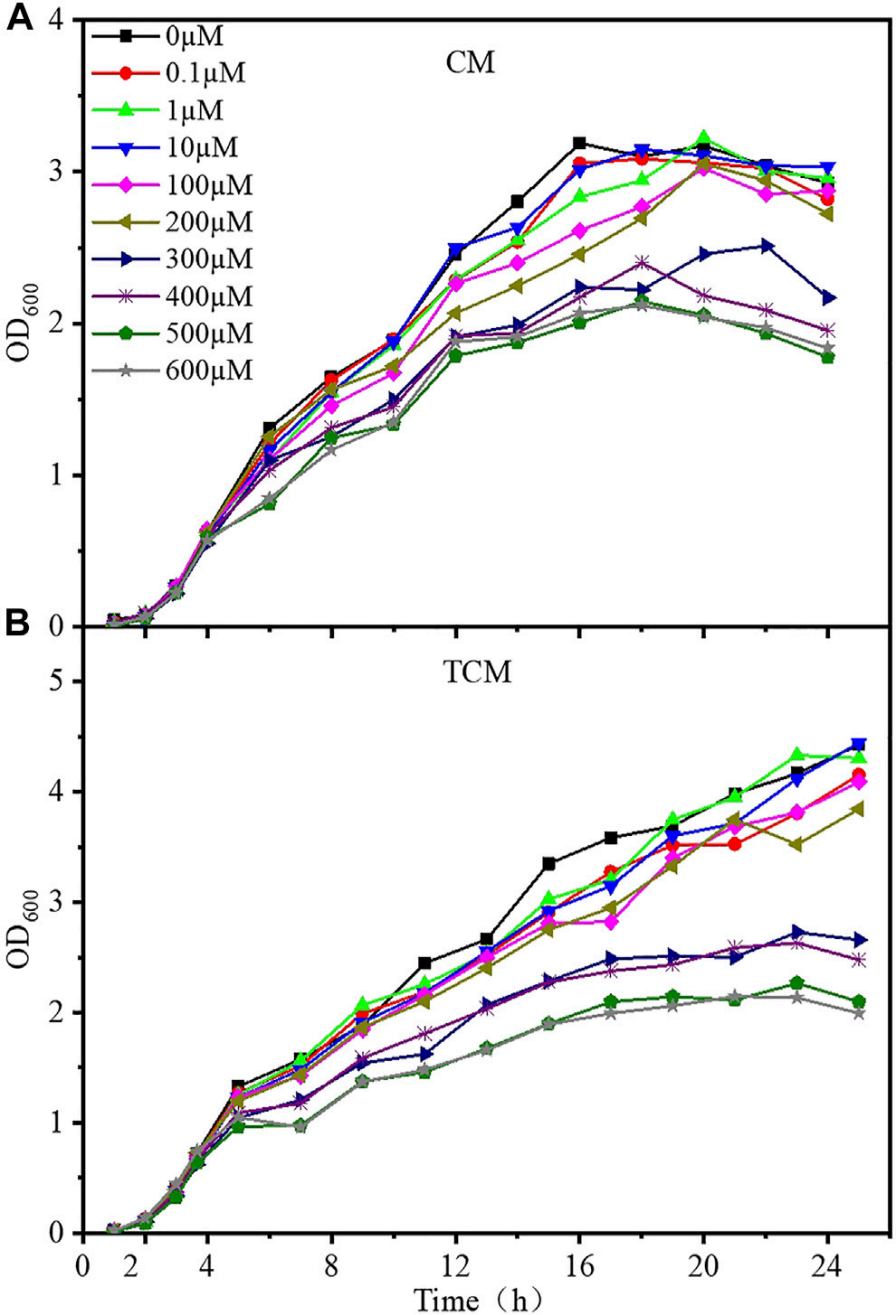
Growth curves of the two WCB sensor strains at various Cd^2+^ concentrations. **(A)** Without negative feedback, WCB CM; **(B)** With negative feedback, WCB TCM.

The growth curves of the CM and TCM sensor strains were evaluated at different doses of cadmium ions to better understand the harmful effects of cadmium on the designed strains. Samples were taken every hour, and Cd^2+^ was added when the OD_600_ value reached 0.6–0.8, after which samples were taken every 2 hours, and the OD_600_ was monitored for 24 h. After around 4 h of incubation, the CM and TCM biosensor strains both entered the logarithmic phase, as shown in Figure 2. The growth of cells was gradually inhibited with the increase of Cd^2+^ concentration. At Cd^2+^ concentrations below 10 μM, the growth of both sensor strains was largely unaffected. Cell growth was inhibited to a lesser extent at Cd^2+^ concentrations of 100–200 μM, and significantly inhibited above 300 μM. From the comparison of the growth curves, it can be concluded that the time to enter the stable phase of the biosensor TCM was delayed at low Cd^2+^ concentrations, and the time in the stable phase was prolonged at high Cd^2+^ concentrations. This indicates that the addition of a negative feedback amplifier improved the tolerance of the biosensor to Cd^2+^. Considering the inhibition of cell growth by Cd^2+^, 0–10 µM Cd^2+^ was selected for subsequent experiments.

### 3.3 Time-dependent Response

**FIGURE 3:**
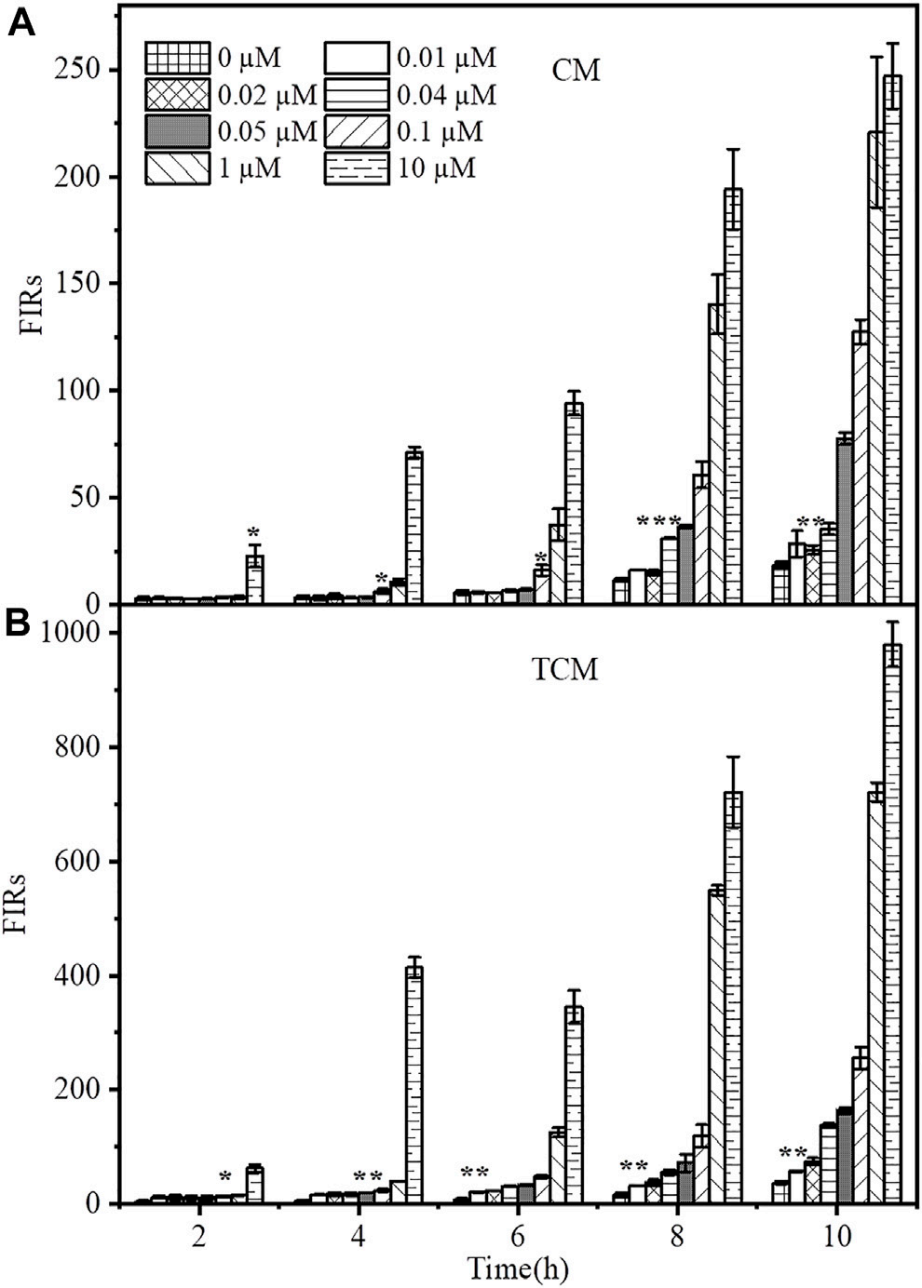
Time-dependent response of Cd^2+^ biosensors without **(A)** and with **(B)** negative feedback. The CM and TCM biosensor cells were grown in medium containing 0, 0.00001, 0.0001, 0.001, 0.01, 0.02, 0.04, 0.05, 0.1, 1, or 10 μM Cd^2+^. The error bars indicate the standard deviation of triplicate experiments. *p < 0.05; **p < 0.01; ***p < 0.001.

In practical applications, the response time of WCBs is a crucial issue to consider. Furthermore, an important factor when using genetic amplifiers in WCBs is base-level expression, stemming either from the sensor or amplification modules. The resulting expression loop can be self-reinforcing, resulting in a significant number of false-positive signals over time. After adding 0, 0.01, 0.02, 0.04, 0.05, 0.1, 1 or10 µM Cd^2+^, the time-dependent responses of the two WCBs were evaluated for 10 h. As shown in Figure 3, both WCBs showed a slight increase in background signal when incubated without Cd^2+^ for longer than 6 h. After 10 h, the fluorescence signal of the negative feedback biosensor TCM increased by about 2.1-fold, while that of the non-positive feedback biosensor CM increased by about 3.25-fold, and the biosensor containing the negative feedback circuit exhibited a reduction of background leakage to some extent. Thus, basal level expression of biosensors with and without negative feedback amplifiers was comparable, with no false-positive signals arising from potential leaky expression.

The fluorescence response of the biosensor gradually increased with time at different Cd^2+^ concentrations (Supplementary Figure S4). After adding Cd^2+^ at concentrations of 0–10 μM and incubating for 8 h, the fluorescence response of the biosensor CM to Cd^2+^ with a concentration of 0.04 μM changed significantly, and its response time was 8 h. TCM produced a significant fluorescence response to 0.05 μM Cd^2+^ at 4 h. Thus, the response time was reduced by 4 h. After 8 h of incubation, TCM produced a significantly higher fluorescence response to 0.01 μM Cd^2+^ than CM, which only produced a significant change at 0.04 μM Cd^2+^. This indicates that the addition of the negative feedback amplifier can significantly reduce the response time of the biosensor. In addition, the negative feedback biosensor TCM responded faster to Cd^2+^ with a much higher signal than the biosensor CM without negative feedback. The fluorescence intensity of TCM was about 3.91 times higher than that of CM after adding Cd^2+^ at a concentration of 0.1 µM for 8 h, and after 4 h of exposure to 10 μM Cd^2+^, its output signal was 5.81 times higher than that of CM. In order to compare the sensitivity, detection limit, and specificity between the sensors TCM and CM under the same condition, we set the incubation time of the sensor TCM the same to the sensor CM, that is 8 h, in the Concentration-dependent fluorescence response and Specificity tests. The minimum detection limit of the sensor TCM is 0.05 μM at 4 h, which does not meet the cadmium detection standard of 0.027 μM in drinking water specified by the WHO; while the minimum detection limit of the sensor TCM is 0.01 μM at 6 h, which does meet the requirement for cadmium determination. Therefore, in Analyses of spiked sample, we stipulated that the response time of the sensor TCM is 6 h.

### 3.4 Concentration-Dependent Fluorescence Response

**FIGURE 4:**
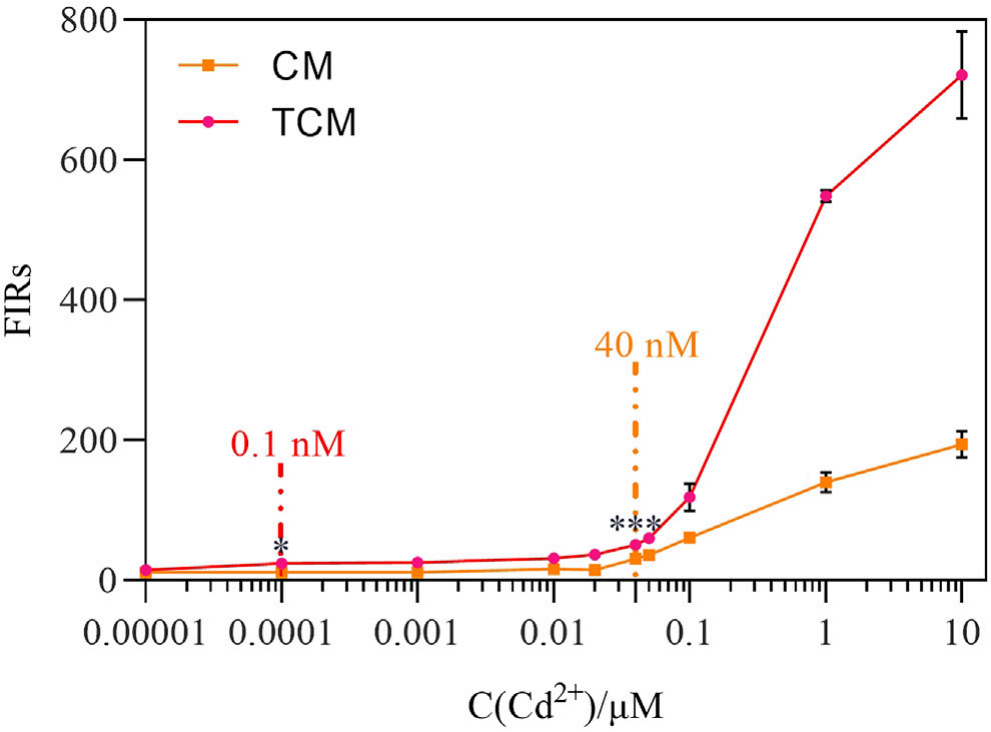
Dose-dependent fluorescence response of the biosensor CM without negative feedback and biosensor TCM with negative feedback. The error bars represent the standard deviations from triplicate experiments. *p < 0.05; **p < 0.01; ***p < 0.001.

Next, the amplification impact of the negative feedback loop was analyzed at different initial Cd^2+^ concentrations. After 8 h of exposure to Cd^2+^ at concentrations ranging from 0 to 10 μM at 30°C, the two WCBs were compared. Both WCBs exhibited a dose-dependent pattern with a positive relationship between fluorescence intensity and Cd^2+^ concentration (Figure 4). At the same time, linear fitting was performed on the fluorescence response data measured at different Cd^2+^ concentrations (Supplementary Figure S5), and the linear response range of the biosensor CM was 0.04–0.1 µM, with a sensitivity of 509.09. By contrast, the linear response range of the biosensor TCM was 0.0001–0.05 µM, and its sensitivity was 678.07. Hence, the latter was 33% more sensitive. The output signal of the WCB with negative feedback was significantly amplified at Cd^2+^ concentrations of 0.00001–10 μM, and was 1.31–3.71 times higher than the output signal of the WCB without negative feedback at the same concentrations. There was detectable MCherry expression when Cd^2+^ was added at a concentration as low as 0.1 nM (*p* < 0.05) for the WCB with negative feedback and 40 nM (*p* < 0.05) for the one without negative feedback. After significance analysis, the detection limit of the biosensor CM at 8 h was 40 nM, and the detection limit of the biosensor TCM was 0.1 nM, which is a 400-fold increase. Notably, the detection limit of the biosensor TCM is significantly lower than the WHO standard for Cd^2+^ detection in drinking water (27 nM). These findings demonstrate that a WCB with a negative feedback amplifier exhibits significantly improved fluorescence intensity, detection range, and sensitivity.

### 3.5 Specificity of the Constructed WCBs

**FIGURE 5:**
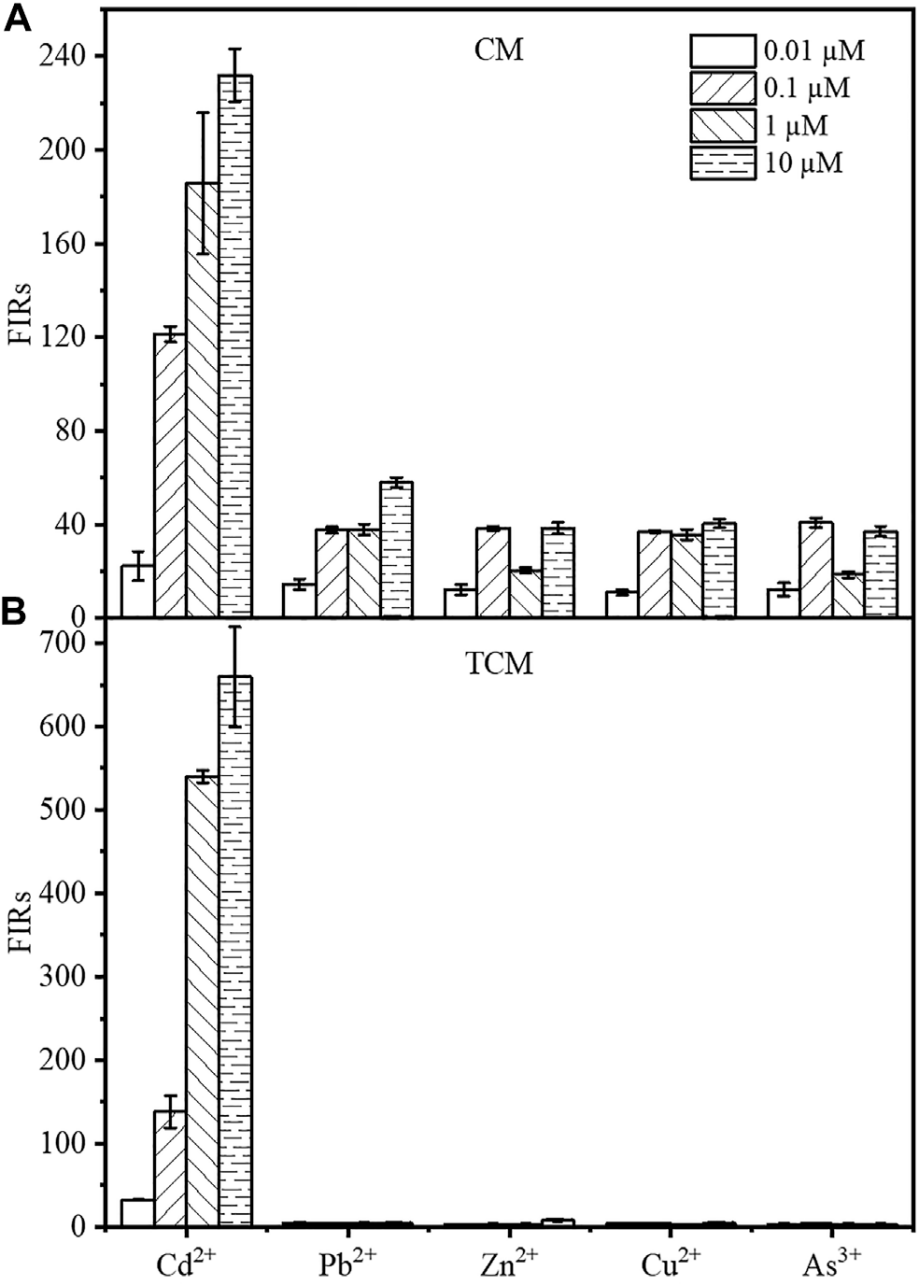
The Cd^2+^ specificity of the biosensor CM without negative feedback **(A)** and the negative feedback amplifier biosensor TCM **(B)**. The fluorescence intensity of the biosensor was measured by incubating the cells for 8 h in the presence of 0.01, 0.1, 1, or 10 µM Cd^2+^, respectively. The error bars represent standard deviations from triplicate experiments.

The specificity for cadmium, in addition to the sensitivity and intensity of the output signal, is an important factor in the evaluation of WCBs. The mCherry fluorescence of the two WCBs was evaluated after 8 h of incubation in the presence of CdCl_2_, Pb(NO_3_)_2_, ZnCl_2_, CuCl_2_, or NaAsO_2_ at final concentrations of 0.01, 0.1, 1, or 10 µM. As shown from Figure 5, the response of the TCM biosensor to other metals was negligible at 1 and 10 μM, with single intensities less than 0.51 or 1.19% compared to Cd^2+^ at the same concentration. At 0.01 µM and 0.1 µM, the signal from other metals was 2.27–13.54% compared to Cd^2+^. By contrast, the fluorescence response of the biosensor CM to other metals was approximately 31–65% at 0.01 and 0.1 µM, 10–20% at 1 μM and 16–25% at 10 µM. The cadmium output signal of the WCB was therefore considerably improved by using a negative feedback amplifier. The specificity for cadmium ions was also considerably improved, which is noteworthy given that no other design has been documented to improve WCB specificity using negative feedback circuit engineering.

### 3.6 Analyses of Spiked Sample

Natural river water containing complex components may influence the specificity and vitality of bacterial biosensors, resulting in incorrect results in some circumstances. Furthermore, complex components in natural water may interfere with the target inducer by rendering it inaccessible to bacteria. The performance of the TCM biosensor was therefore evaluated using a series of artificially cadmium-contaminated river water samples. Inductively coupled plasma-mass spectrometry (ICP-MS) analysis determined that the cadmium concentration was 0.00054 μM in the original river water samples. The Cd^2+^ calibration curve of the TCM biosensor was established after 6 h of induction at final Cd^2+^ concentrations 0.01, 0.02, 0.04, and 0.05 μM. Cadmium concentrations in spiked cadmium-contaminated river water samples were estimated using equation Y = 18.10713 + 294.92341 × X. As can be seen in Table 1, the recovery rates of cadmium determined by the TCM biosensor ranged from 98.08 to 101.11%, which corresponded well to the actual concentrations of the samples. However, high concentration of heavy metal ions may exceed the tolerance of the sensor and then affect its detection ability. Therefore, we also tested the performance of this WCB at higher Cd^2+^ concentrations such as 5, 10, 50, 100, and 200 μM, with the recovery rates of 97.18, 97.65, 102.74, 104.32, and 95.45%, respectively (Supplementary Table S2). These findings suggest that the biosensor TCM can achieve quantitative detection of cadmium in a low concentration range and has a high level of interference resistance and stability for detecting medium and high concentration cadmium in river water samples.

**TABLE 1:** Accuracy and reliability of the constructed TCM biosensor in the measurement of cadmium in real river water samples.

Samples	ICP-MS (μM)	Cd^2+^ added (μM)	FIRs^a^	Estimated (μM)	Recovery (%)
River water	0.00054	0.01	21.21 ± 0.52	0.01066	101.11
	0.00054	0.027^b^	26.01 ± 0.55	0.02727	99.02
	0.00054	0.045^c^	31.24 ± 0.66	0.04467	98.08
	0.00054	0.05	33.28 ± 0.95	0.05101	100.92

a Mean FIR, value ± standard deviation (*n* = 3).

b Mean 0.027 μM is the legal limit for cadmium ions in drinking water set by the WHO (WHO, 2011).

c Mean 0.045 μM is the legal limit for cadmium ions specified in the national sanitary standard of China for domestic drinking water (GB 5749-2006) (Jin et al., 2006).

## 4 Discussion

The ability of WCBs to detect harmful heavy metals and metalloids (such as cadmium, lead, mercury and arsenic) in the environment has been extensively studied (Wang et al., 2013; Wan et al., 2019; He et al., 2021). Despite the fact that many WCBs have been developed and used for metal ion detection, most WCBs cannot be used for environmental monitoring because they do not meet the high sensitivity and specificity requirements (Hansen and Sørensen, 2001; Baronian, 2004; Guo et al., 2019). Pola-López et al. created a novel vector in which phage T7 RNA polymerase serves as an amplifier and green fluorescent protein (GFP) serves as a reporter protein (Pola-López et al., 2018). The biosensor they designed has a detection range of 5–140 μg/L, and concentrations of the metalloid As(III) below the WHO limit were successfully detected. However, in contrast to our design, the amplifier did not improve the performance of the arsenic biosensor, and a biosensor without the amplifier was not used as a control to evaluate the amplification effect.

In order to improve the output signal and boost the sensitivity of WCBs, many studies incorporated genetic amplifier circuits (Nistala et al., 2010; Jia et al., 2018). However, negative feedback loops have a different role in enhancing loop sensitivity depending on the genetic context and regulatory factors present. Savageau made the early assertion that “negative feedback regulation is more stable than open-loop or positive feedback regulation” (Savageau, 1974). TetR negative feedback loops have been used to regulate a variety of protein-coding genes to increase output signals or improve sensitivity. However, their use for Cd^2+^ detection has not been reported (Bertram and Hillen, 2008; Wang et al., 2015c). Our study shows that the TetR negative feedback circuit coupled with the cadR regulation circuit not only improves the sensitivity of the biosensor but also improves the selectivity of the biosensor for Cd^2+^.

One problem with adding amplifiers to WCBs is the high noise level and the false positive signal amplified due to basal expression and promoter leakage. While this leakage can be negligible in some application contexts, it can cause problems in other situations. For example, 1) at high basal expression, sensitive enzyme-based colorimetric outputs can rapidly saturate, limiting titrimetric analysis (Wackwitz et al., 2008); 2) reducing the dynamic range of the downstream output reporter expression (Nielsen et al., 2016); and 3) causing non-severe side effects in biosensor outputs with therapeutic killing capabilities. In addition, negative feedback plays an essential role in achieving bistable gene expression, traditionally referred to as high/low or ON/OFF. In these scenarios, the initial concentration of the inducer can determine the gene expression level of the biosensor, and the initial input level of the inducer can induce a switch in the gene expression of the biosensor between two steady states. Single-cell studies revealed that the activity of the TetR protein determined whether the negative feedback amplifier enhanced cell noise relative to the non-negative feedback control. Both the negative feedback and non-negative feedback systems have comparable noise levels. At greater inducer concentrations, however, the increase in noise may be attributed to an increasing system burden caused by slower cell growth rates. In our work, to compare the basal background of the biosensors CM and TCM, we measured the time-dependent response of the two biosensors at different Cd^2+^ concentrations. We found that the basic expression levels of the biosensors with and without negative feedback amplifiers were comparable. False-positive signals generated by potential amplification of leaky expression were not observed, and the biosensor TCM with a negative feedback circuit reduced background leakage to a certain extent.

Sensitivity and specificity are the primary indicators of microbial biosensor performance, with sensitivity being related to the detection limit as well as the resolution, and specificity being the ratio of the signal intensity of the response to the target detection to that of other ions. In 2015, Hou et al. constructed the sensor pcadCluc and pzntRluc for Cd^2+^ and Pb^2+^ with detection limits below 0.1 and 0.05 μM, respectively, and the sensor parsRluc for Cd^2+^ with a detection limit of 5.0 μM and less than 0.1 μM for As^3+^ (Hou et al., 2015). In 2015, Kumar et al. developed a microarray-based synthetic system biosensor, which reduced the reaction volume to 200 μL and the detection limit for cadmium was 0.045 μM (Kumar et al., 2017). None of these sensors meet the WHO standard of 0.027 μM for cadmium in drinking water. Since the negative feedback loop can amplify the output signal at a low concentration of Cd^2+^ and improve the sensitivity, the Cd^2+^ biosensor with the negative feedback amplifier had a wider detection range and lower detection limit. The constructed negative feedback biosensor TCM was able to detect cadmium down to 0.0001 µM and was more sensitive than other biosensors (Supplementary Table S3) constructed to date (Sayut et al., 2006; Close et al., 2012). The detection limit of the negative feedback biosensor TCM was 400-fold higher than that of the negative feedback-free biosensor CM.

In 2017, Biosensors and Bioelectronics published a paper on the functional study of a composite microbial cadmium biosensor based on different genera of Gram-negative bacteria develop by Franks’ team at La Trobe University, Australia. They expressed a multiplex cadmium microbial sensor in different Gram-negative bacteria. However, the detection limits of Cd^2+^ did not meet the Chinese standard of cadmium concentration in drinking water (45 nM or 0.005 mg/L), and the multiple cadmium microbial biosensor responded to several other heavy metals (e.g., similar concentrations of arsenic, mercury, and lead) (Bereza-Malcolm et al., 2017). In 2018, the sensor developed by the Bereza-Malcolm group expressed in different strains, exhibited better selectivity in the detection of arsenic ions, but could not completely overcome the interference problem of other heavy metal ions, e.g., the response signal to arsenic ions at 100 μg/L was only three times higher than that of other metal ions (Bereza-Malcolm et al., 2018). In addition to increasing the biosensor’s sensitivity, the specificity of the negative feedback amplification system for Cd^2+^ was also improved, since it significantly amplified the response of the fluorescence signal to Cd^2+^, while the response to other metals was only slightly increased. As a result, it exhibited an outstanding fluorescence difference in response to CdCl_2_ and other metals in general. Many cadmium WCBs have specificity issues (Sayut et al., 2006; Close et al., 2012; Segall-Shapiro et al., 2014; Kim et al., 2016; Yoon et al., 2016; Bereza-Malcolm et al., 2017). In general, protein engineering can enhance specificity by changing the interplay between regulatory factors and induced metal ions. Surprisingly, the negative feedback loop added to cadmium WCBs selectively increased the fluorescence output signal in response to cadmium and other metals. The fluorescence response of the sensor at a Cd^2+^ concentration of 0.1 µM is 34.2 times, 17.6 times, 34.6 times, and 41.4 times that of 10 μM Pd^2+^, Zn^2+^, Cu^2+^, As^3+^, respectively (Figure 5B). At 0.01, 0.1, 1, 10 µM concentration gradients, the signal produced by Cd^2+^ was between 7 and 197 times stronger than the fluorescence output signals of other metals, allowing the accurate detection of cadmium in the presence of other metals. This may be because the specific promoter P_*lteto1*_ regulates the expression level of *cadR* gene. At the same time, aTc molecules diffuse into the cytoplasm and bind to TetR, somewhat alleviating its repression of the specific promoter P_*lteto1*_, which results in the expression of the metal-binding protein CadR at an appropriately lower concentration in the biosensor TCM compared with the biosensor CM without the negative feedback amplifier. The special metal-binding sites of cadmium-responsive transcription factor CadR protein determine their different binding abilities to certain metals. Therefore, when cadmium is present simultaneously with other metal ions, the biosensor CM without negative feedback amplifier will bind metal ions with a weaker binding capacity to CadR to a large extent, after preferentially binding cadmium due to the higher intracellular CadR concentration. On contrary, the biosensor TCM containing negative feedback amplifier expresses the CadR at an appropriately low concentration, which can highly bind to cadmium and increase the specificity of the sensor.

The difference between the actual measurement results and the initial concentration of the sample is a worrying problem in practical applications, because when a water sample is added to the medium, the analyte will be diluted. Even if the initial concentration is within the detection limit of the sensor, a high dilution ratio may cause the sample to be undetectable. One method is to make Luria-Bertani (LB) broth with sample water and use it to inoculate WCBs. Since bacteria, like many other WCBs (Hansen and Sørensen, 2001), do not require special conditions for rapid growth and reproduction, we chose bacteria as the host in this study. Bacteria can not only survive well at room temperature, but they also have relatively low requirements in terms of pH and humidity. Bacterial WCBs are relatively more robust because the expression of reporter proteins is dependent on cell growth. The Cd^2+^ concentration determined by the TCM biosensor closely matched the actual concentration of the sample, showing strong resistance to interference and good stability. Although the current biosensor cells require 6 h of incubation to produce sufficient fluorescence output for the actual assay, they can be further optimized to reduce the processing time if needed. For example, the cell density can be optimized to increase the fluorescence level so that the output can quickly reach the visual detection threshold, and the output based on a fast enzymatic reaction (for example, NanoLuc (Cevenini et al., 2018) or LacZα peptide (Ma et al., 2018) can accelerate the detection response. Compared with the incubation time of WCBs under other real sample detection conditions (Supplementary Table S4), the sensor TCM with negative feedback amplifier can achieve the quantitative detection of cadmium in the low concentration range of 0.01–0.05 µM (which can achieve WHO drinking water cadmium detection standard of 0.027 μM) in 6 h. This research demonstrates the modulation element’s sensitivity in Cd^2+^ detection, as well as the role of negative feedback in improving the sensitivity. In order to avoid problems caused by using living cells as hosts, the same regulatory elements can also be used in the design of cell-free Cd^2+^ biosensors.

## 5 Conclusion

Overall, in terms of reaction time, sensitivity, and specificity, our findings reveal that the negative feedback biosensor TCM outperforms the non-negative feedback biosensor CM. This biosensor with a negative feedback amplifier is capable of detecting cadmium ions below WHO and FAO standards. It has the potential to be used as a field detection tool for regular monitoring of cadmium levels in drinking water. Our research emphasizes the significance of genetic circuit engineering in enhancing the performance of WCBs and provides new ideas for the development of additional biosensors. Negative feedback loops are a workable strategy to construct and improve WCBs for the detection of other heavy metals or pollutants, even though their influence on negative feedback circuits may vary due to genetic background.

## Data Availability

The original contributions presented in the study are included in the article/Supplementary Material, further inquiries can be directed to the corresponding author.
