# Supplementary material for: Highly Sensitive Whole-Cell Biosensor for Cadmium Detection Based on a Negative Feedback Circuit
Source: Front Bioeng Biotechnol. 2021 Dec 3;9:799781. doi: 10.3389/fbioe.2021.799781 (PMC8678453; doi:10.3389/fbioe.2021.799781)
Supplement: Supplementary file 1 [file DataSheet1.docx]

**Table S1** List of genetic parts and sequences used in this study (RBSs are in italic and bold)

| Part name | \| DNA sequence (5’–3’) \| \| --- \| |
| --- | --- | --- |
| P*_lteto1_* | TTTTCAGCAGGACGCACTGACCTCCCTATCAGTGATAGAGATTGACATCCCTATCAGTGATAGAGATACTGAGCACATAT |
| P*_cad_* | CACGAAATCTCCAGCAAGTGGCTTGACCCTATAGTGGCTACAGGGTGTTCACTTGGCAACAGGCTCAATTTAAGGATGACCCC |
| RBS-TetR | ***ATACGTATTTAAATCAGGAGTGGAA***ATGAGCCGTCTGGACAAGAGCAAGGTGATCAACAGCGCCCTGGAACTGCTGAACGAGGTGGGCATCGAGGGCCTGACCACCCGCAAGCTGGCCCAGAAGCTGGGCGTCGAACAGCCGACCCTGTACTGGCACGTGAAGAACAAGCGTGCCCTGCTGGACGCCCTGGCCATCGAGATGCTGGACCGCCATCACACCCATTTCTGCCCACTGGAAGGCGAGAGCTGGCAGGACTTCCTGCGCAACAACGCCAAGAGCTTCCGCTGCGCCCTGCTGAGCCATCGTGACGGTGCCAAGGTCCACCTGGGCACCCGTCCGACCGAGAAGCAGTACGAAACCCTGGAAAACCAGCTGGCCTTCCTGTGCCAGCAGGGTTTCTCGCTGGAAAACGCCCTGTACGCCCTGAGCGCCGTGGGCCATTTCACCCTGGGCTGCGTGCTGGAAGATCAAGAGCACCAGGTGGCCAAAGAGGAACGCGAAACCCCGACCACCGACAGCATGCCACCGCTGCTGCGCCAGGCCATCGAACTGTTCGACCACCAGGGTGCCGAGCCAGCGTTCCTGTTCGGTCTGGAACTGATCATCTGCGGTCTGGAAAAGCAACTGAAGTGCGAAAGCGGCAGCTGA |
| RBS-cadR | ***ATACGTATTTAAATCAGGAGTGGAA***ATGAAGATCGGAGAACTGGCCAAAGCCACCGACTGCGCGGTGGAAACCATCCGCTACTACGAGCGTGAAAACCTGCTGCCAGAGCCGGCGCGCAGCGAGGGCAACTACCGGTTGTACACCCAGGCCCATGTGGAGCGGCTGACCTTCATCCGCAACTGCCGCACGCTGGACATGACCCTGGACGAAATTCGCAGCCTGCTACGCCTGCGCGACAGCCCCGACGACGCGTGCGGCAGCGTCAATGCGCTGATCGACGAGCATATCGAGCATGTTCAGGCGCGGATCGATGGCTTGGTGGCATTGCAGGAGCAGCTGGTGGAGCTGCGGCGGCGCTGCAACGCGCAGGGGAGTGAATGCGCGATCTTGCAGCAACTGGAGACAAACGGGGCGGTATCGGTACCGGATACCGAACATTCCCATGTGGGGCGGAGTCACGGGCATTGA |
| RBS-mCherry | ***ATACGTATTTAAATCAGGAGTGGAA***ATGGTGAGCAAGGGCGAAGAGGACAACATGGCCATCATCAAAGAGTTCATGCGCTTCAAGGTGCACATGGAAGGCAGCGTGAACGGCCACGAGTTCGAGATCGAAGGCGAAGGCGAGGGTCGTCCGTACGAGGGCACCCAGACCGCCAAGCTGAAGGTGACCAAAGGCGGTCCGCTGCCGTTCGCCTGGGACATCCTGTCGCCACAGTTCATGTACGGCAGCAAGGCCTACGTGAAGCACCCAGCGGACATCCCGGACTACCTGAAGCTGAGCTTCCCGGAAGGCTTCAAGTGGGAGCGCGTGATGAACTTCGAGGACGGTGGCGTGGTGACCGTGACCCAGGACAGCAGCCTGCAGGACGGCGAGTTCATCTACAAGGTGAAGCTGCGTGGCACCAACTTCCCGAGCGACGGTCCGGTGATGCAGAAAAAGACCATGGGCTGGGAAGCCAGCAGCGAGCGCATGTACCCGGAAGATGGTGCCCTGAAGGGCGAGATCAAGCAGCGCCTGAAACTGAAGGATGGCGGTCACTACGACGCCGAGGTCAAGACCACCTACAAGGCCAAGAAGCCGGTCCAGCTGCCAGGTGCCTACAACGTGAACATCAAGCTGGACATCACCAGCCACAACGAGGACTACACCATCGTGGAACAGTACGAGCGTGCCGAAGGCCGTCACAGCACCGGTGGCATGGACGAGCTGTACAAGTGA |
| BBa_B0015 | TAATACTAGAGCCAGGCATCAAATAAAACGAAAGGCTCAGTCGAAAGACTGGGCCTTTCGTTTTATCTGTTGTTTGTCGGTGAACGCTCTCTACTAGAGTCACACTGGCTCACCTTCGGGTGGGCCTTTCTGCGTTTATA |

**Table S2** Accuracy and reliability of the constructed biosensor TCM in the measurement of medium and high concentration cadmium in real river water samples

| **Cd^2+^ added (μM)** | **FIRs^α^** | **FIRs****^β^** | **Recovery rate^λ^** |
| --- | --- | --- | --- |
| 5 | 50.32±4.82 | 48.90±5.43 | 97.18%±1.50% |
| 10 | 94.09±5.58 | 91.88±4.47 | 97.65%±1.04% |
| 50 | 284.32±18.32 | 292.11±20.45 | 102.74%±0.57% |
| 100 | 473.56±20.22 | 494.02±22.22 | 104.32%±0.23% |
| 200 | 723.42±28.67 | 690.50±30.52 | 95.45%±0.42% |

Note: ^α^ Mean cadmium concentration-dependent fluorescence response when LB was prepared with distilled water. ^β^ Mean cadmium concentration-dependent fluorescence response when LB was prepared with river water. **^λ^** Mean recovery rate = FIRs^β^/FIRs^α^. Each experiment was repeated three times, and the error was expressed in standard deviation.

**Table S3** Comparison of WCBs’ limit of detection under laboratory conditions

| **Microbial biosensor** | **Gene circuit** | **Sample type and limit of detection** | **Reference** |
| --- | --- | --- | --- |
| *P. putida* KT2440 (TCM)^a^ | P_lteto1_-cadR-tetR-ter-P_cad_-mcherry-ter | Cd^2+^: 0.0001 µM | Present study |
| *E. coli* DH5ɑ (pVLCD1)^a^ | P*_cadC_*-*cadC*-*gfp* | Cd^2+^: 0.0001 µM；Pb^2+^: 0.001 µM; Sb^3+^: 0.0001 µM | (Liao et al., 2006) |
| *E. coli* DH5ɑ  (pcadCluc/pzntRluc)^a^ | P*_cadA_*-*cadC*-*luc*；  P*_nptII_*-*zntR*-TrrNB-P*_znt_*-*luc* | Cd^2+^: 0.1 µM; Pb^2+^: 0.05µM | (Hou et al., 2015) |
| *E. coli* DH5α/*P.aeruginosa* PAO1/*S.oneidensis* MR-1/*Enterobacter spp.* NCR3 and LCR17 (pBB*cadRgfp*-*rfp*)^a^ | *mrf*p1-*ter*-*cadR*-P*_cad_*-*gfp* | Cd^2+^: 17.8 µM (DH5α) / 0.89 µM (PAO1) / 88.96 µM (MR-1) / 2.224 µM (NCR3) / 8.896 µM (LCR17) | (Bereza-Malcolm et al., 2017a) |
| *P. putida* KT2440 (p2T7RNAPmut-68)^a^ | *cadR*-P*_cad_*-RBS-*T7RNAP*  + P*_T7_-cadO-*RBS-*mcherry* | Cd^2+^: 0.01 µM | (Jia et al., 2021) |
| *Escherichia coli* MG1655k12 (POLA (ABP))^b^ | P*_arsR_*-RBS-*T7RNAP-ter-*P*_T7_-*RBS*-gfp-ter* | As^3+^: 0.0668 µM | (Pola-López et al., 2018) |
| *E. coli* DH5α (pNV12)^c^ | P*_cadC_*-*cadC*-*gfpmut3*α | Cd^2+^: 0.045 µM, microarray | (Kumar et al., 2017) |
| *P. putida* KT2440/KT2440.2431 (pDNPczc1lux)^d^ | P*_czc1_*-*czcCBA1*-*luxCDABE* | Cd^2+^: 1.12 µM (KT2440) / 0.09 µM (KT2440.2431) | (Hynninen et al., 2010) |
| *P. putida* 06909 (pVPLGMPC)^e^ | P*_tac_*-*cadR*-*ter*-P*_cadR_*-*lacI*^q^-*gfp* | Cd^2+^: 0.01 µM | (Wu et al., 2009) |
| *E. coli* TOP10 (pNTCOG / pNTCOG-TC10 / pNTCOG-TC21)^f^ | *gfp*-P*_cadR_*-*cadr-x* | Cd^2+^: 2.0 µM (COG) / 1.3 µM (COG-TC10) / 1.5 µM (COG-TC21); Zn^2+^: 1.1 µM (COG)/0.9 µM (COG-TC10) / 1.2 µM (COG-TC21); Hg^2+^: 1.0 µM (COG) / 1.6 µM (COG-TC10) / 3.8 µM (COG-TC21) | (Tao et al., 2013) |

Note: ^a^ The detection limit is determined by the statistically significant change (P<0.05) compared with the background value; ^b^ the fluorescence grew linearly with elapsed time as the arsenite increased; ^c^ the detection limit is the Cd^2+^ concentration that caused an obvious response (background + 3×standard deviation); ^d^ the detection limit is the metal concentration whose fluorescence response is higher than the threshold level of LOD_IC_, LOD_IC_=(1+2*CV_w_)/(1-2*CV_w_), where CVw is the signal change coefficient measured in water (background or blank). The equation guarantees that when the confidence interval is 97.7%, the lowest signal L_M_-2*SD_M_ from the sample is higher than the highest value L_W_+2*SD_W_ of the blank sample with the same confidence interval; ^e^ the detection limit is defined as the lowest Cd^2+^ response concentration, where GFP expression is greater than the upper limit of standard error of GFP expression in background samples; ^f^ the detection limit is defined as *IC_LOD_=(Fb+3*SD)/Fb*, where *Fb* is the average background fluorescence value of the sensor without Cd^2+^, and *SD* is the standard deviation.

**Table S4** Comparison of WCBs’ incubation time under real samples detection conditions

| **Microbial biosensor** | **Incubation time (h)** | **Sample type and limit of** **detection (µM)** | **Reference** |
| --- | --- | --- | --- |
| *P. putida* KT2440 (TCM)^α^ | 6 | Cd^2+^: 0.01-0.05 | Present study |
| *E. coli DH5α* (ParsD-ABS-8)^β^ | 4 | As^3+^: 0.1-4 | (Chen et al., 2019) |
| *Enterobacter spp.* NCR3 (pBB*cadRgfp*-*rfp*)^β^ | 7 | Cd^2+^: 4.45, 8.90, 44.48 | (Bereza-Malcolm et al., 2017b) |
| *E. coli* EK317 (CadR.10Z)^α^ | 8 | Cd^2+^: 0−0.2 | (He et al., 2021) |
| *E. coli* TOP10 (As0−3 and As5)^β^ | 24 | As^3+^: 0.013, 0.13,  0.67, 1.33 | (Wan et al., 2019) |

Note: ^α^ The biosensor can achieve the detection of the World Health Organization drinking water cadmium detection standard of 0.027 μM.

**
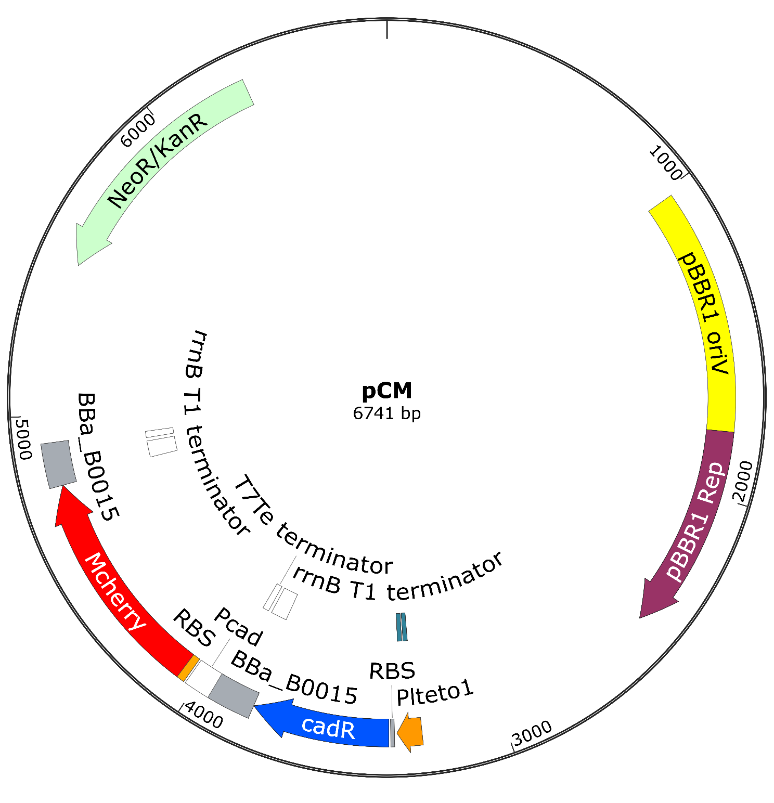
Fig. S1** Schematic diagram of the construction of recombinant plasmids pCM. Cadmium WCB CM gene circuit was constructed in the broad-host shuttle plasmid pBBR1MCS-2.

**
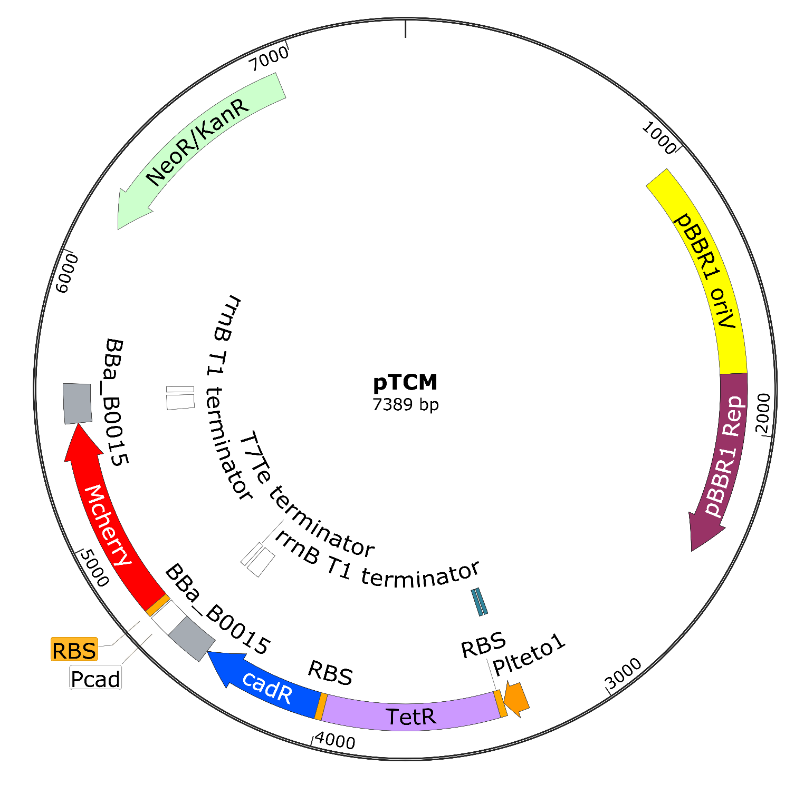
Fig. S2** Schematic diagram of the construction of recombinant plasmids pTCM. Cadmium WCB TCM gene circuit was constructed in the broad-host shuttle plasmid pBBR1MCS-2.


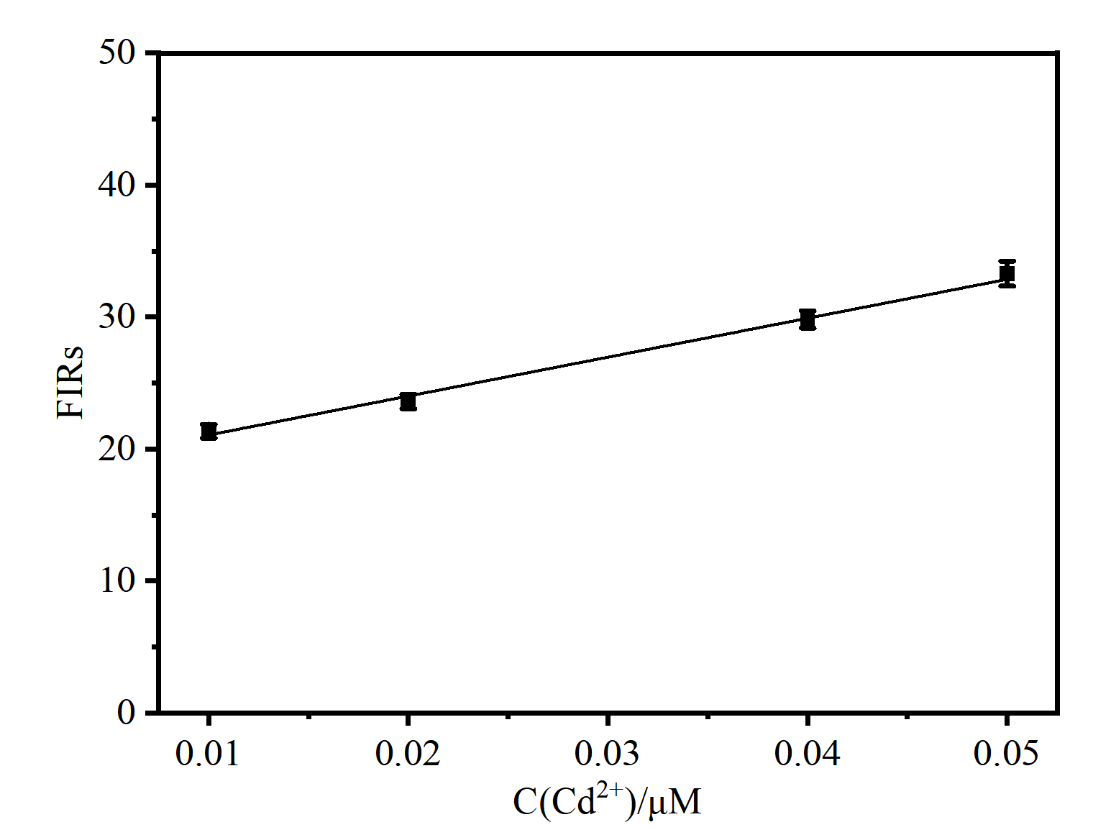
**Fig. S3** Calibration curve of cadmium with concentration range from 0.01 to 0.05 μM. Average fluorescence of each sample was achieved after induction for 6 h at 25°C. Each experiment was repeated three times and standard deviations were shown as error bars.


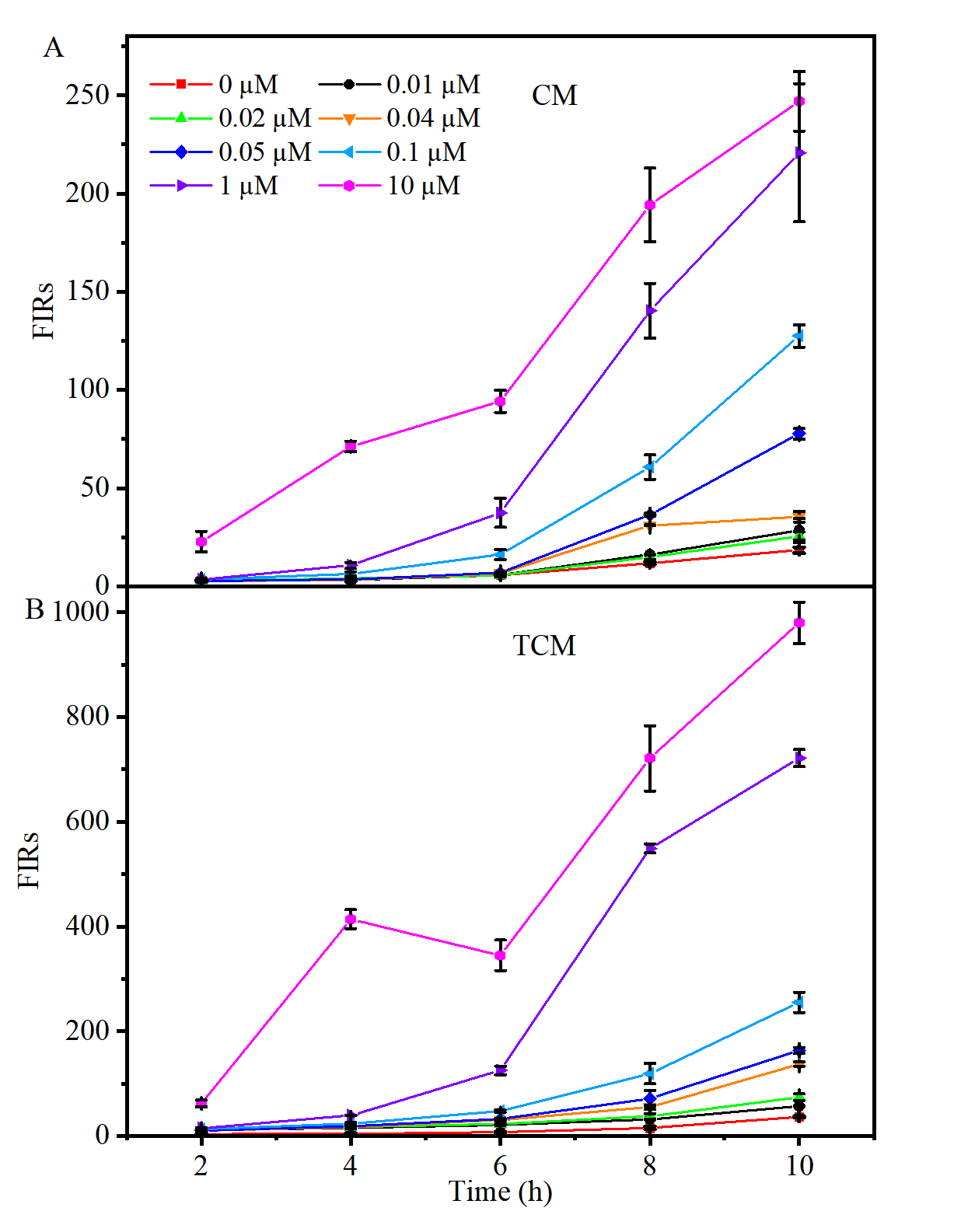
**Fig. S4** Dose-response curves for different time of Cd^2+^ biosensors without **(A)** and with **(B)** negative feedback. The CM and TCM biosensor cells were grown in medium containing 0, 0.00001 μM, 0.0001 μM, 0.001 μM, 0.01 μM, 0.02 μM, 0.04 μM, 0.05 μM, 0.1 μM, 1 μM, or 10 μM Cd^2+^. The error bars indicate the standard deviation of triplicate experiments.


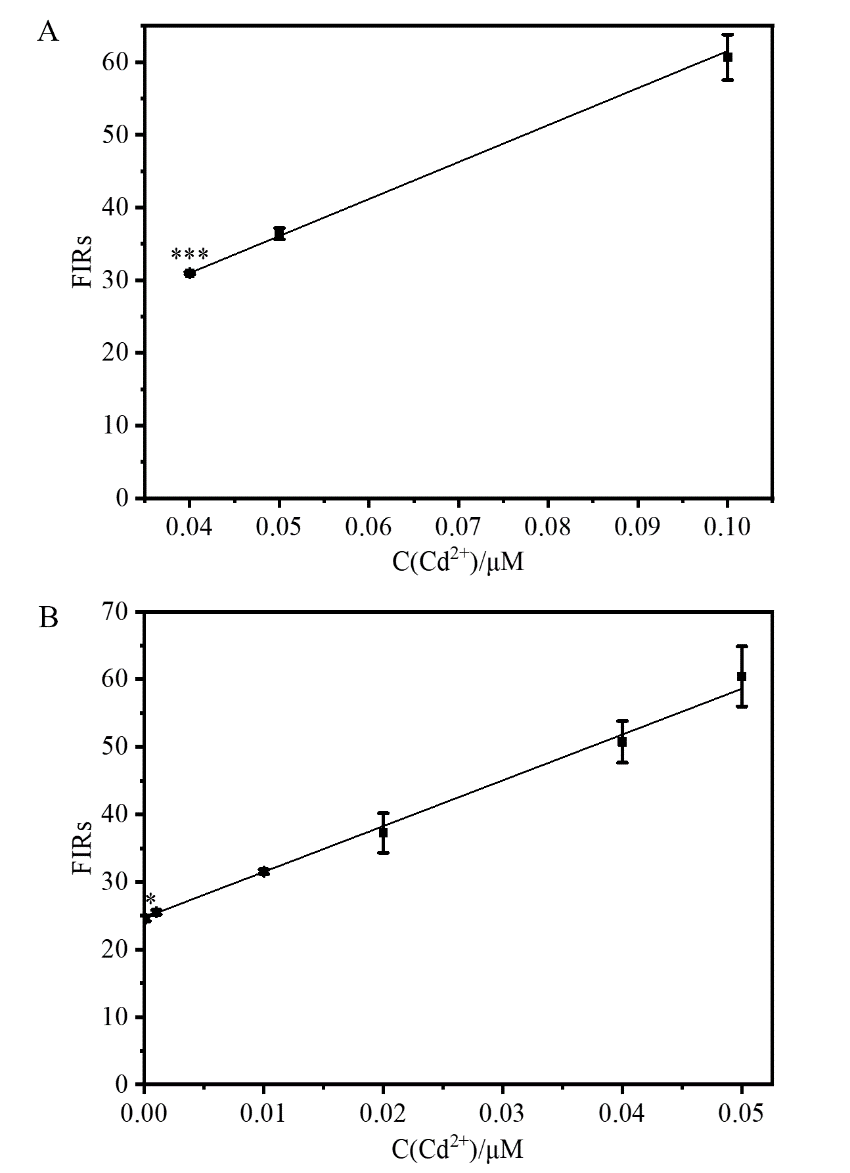
**Fig. S5** The linear fit of the fluorescence response data of the sensor CM (A) and TCM (B) to different Cd^2+^ concentrations. The error bars indicate the standard deviation of triplicate experiments.

Bereza-Malcolm, L., Aracic, S., Kannan, R., Mann, G., and Franks, A.E. (2017a). Functional characterization of Gram-negative bacteria from different genera as multiplex cadmium biosensors. *Biosensors & Bioelectronics.* 94, 380-387. doi: 10.1016/j.bios.2017.03.029

Bereza-Malcolm, L., Aracic, S., Kannan, R., Mann, G., and Franks, A.E. (2017b). Functional characterization of Gram-negative bacteria from different genera as multiplex cadmium biosensors. *Biosensors and Bioelectronics.* 94, 380-387. doi: 10.1016/j.bios.2017.03.029

Chen, S.Y., Wei, W., Yin, B.C., Tong, Y., Lu, J., and Ye, B.C. (2019). Development of a Highly Sensitive Whole-Cell Biosensor for Arsenite Detection through Engineered Promoter Modifications. *ACS Synth Biol.* 8, 2295-2302. doi: 10.1021/acssynbio.9b00093

He, M.-Y., Lin, Y.-J., Kao, Y.-L., Kuo, P., Grauffel, C., Lim, C., et al. (2021). Sensitive and specific cadmium biosensor developed by reconfiguring metal transport and leveraging natural gene repositories. *ACS sensors.* 6, 995-1002. doi: 10.1021/acssensors.0c02204

Hou, Q., Ma, A., Wang, T., Lin, J., Wang, H., Du, B., et al. (2015). Detection of bioavailable cadmium, lead, and arsenic in polluted soil by tailored multiple *Escherichia coli* whole-cell sensor set. *Analytical and Bioanalytical Chemistry.* 407, 6865-6871. doi: 10.1007/s00216-015-8830-z

Hynninen, A., Tonismann, K., and Virta, M. (2010). Improving the sensitivity of bacterial bioreporters for heavy metals. *Bioengineered bugs.* 1, 132-138. doi: 10.4161/bbug.1.2.10902

Jia, X.Q., Liu, T., Ma, Y.B., and Wu, K. (2021). Construction of cadmium whole-cell biosensors and circuit amplification. *Applied Microbiology and Biotechnology.* 105, 5689-5699. doi: 10.1007/s00253-021-11403-x

Kumar, S., Verma, N., and Singh, A.K. (2017). Development of cadmium specific recombinant biosensor and its application in milk samples. *Sensors and Actuators B-Chemical.* 240, 248-254. doi: 10.1016/j.snb.2016.08.160

Liao, V.H.C., Chien, M.T., Tseng, Y.Y., and Ou, K.L. (2006). Assessment of heavy metal bioavailability in contaminated sediments and soils using green fluorescent protein-based bacterial biosensors. *Environmental Pollution.* 142, 17-23. doi: 10.1016/j.envpol.2005.09.021

Pola-López, L., Camas-Anzueto, J., Martínez-Antonio, A., Luján-Hidalgo, M., Anzueto-Sánchez, G., Ruíz-Valdiviezo, V., et al. (2018). Novel arsenic biosensor “POLA” obtained by a genetically modified *E. coli* bioreporter cell. *Sensors Actuators B: Chemical.* 254, 1061-1068. doi: 10.1016/j.snb.2017.08.006

Tao, H.-C., Peng, Z.-W., Li, P.-S., Yu, T.-A., and Su, J. (2013). Optimizing cadmium and mercury specificity of CadR-based *E.coli* biosensors by redesign of CadR. *Biotechnology Letters.* 35, 1253-1258. doi: 10.1007/s10529-013-1216-4

Wan, X.Y., Volpetti, F., Petrova, E., French, C., Maerkl, S.J., and Wang, B.J. (2019). Cascaded amplifying circuits enable ultrasensitive cellular sensors for toxic metals. *Nature Chemical Biology.* 15, 540-+. doi: 10.1038/s41589-019-0244-3

Wu, C.H., Le, D., Mulchandani, A., and Chen, W. (2009). Optimization of a whole-cell cadmium sensor with a toggle gene circuit. *Biotechnology Progress.* 25, 898-903. doi: 10.1002/btpr.203

**Supplementary References**
